# Supplementary material for: Structured experience shapes strategy learning and neural dynamics in the medial entorhinal cortex
Source: Res Sq. 2025 May 28:rs.3.rs-6658028. Preprint. [Version 1] doi: 10.21203/rs.3.rs-6658028/v1 (PMC12154143; doi:10.21203/rs.3.rs-6658028/v1)
Supplement: 1 [file NIHPPrs6658028v1-supplement-1.pdf]

Supplementary Materials

Table of Contents

- **Figure S1: S/FT RNNs more robustly solve the tDNMS task compared to NS RNNs.**
- **Figure S2: Shaping RNNs constrains network activity to follow smoother state space trajectories.**
- **Figure S3: Dynamical motifs reveal how training approach affects RNNs' strategy.**
- **Figure S4: Additional analysis on LS error trials.**
- **Figure S5: SL/FT networks fail to learn critical abstractions about trial structure.**
- **Figure S6: Animals learn shared time-coding axes across trial types—mirroring the dynamics predicted by RNNs.**
- **Figure S7: Dynamical motifs make predictions about how RNNs learned strategy extend to novel temporal contexts.**

a

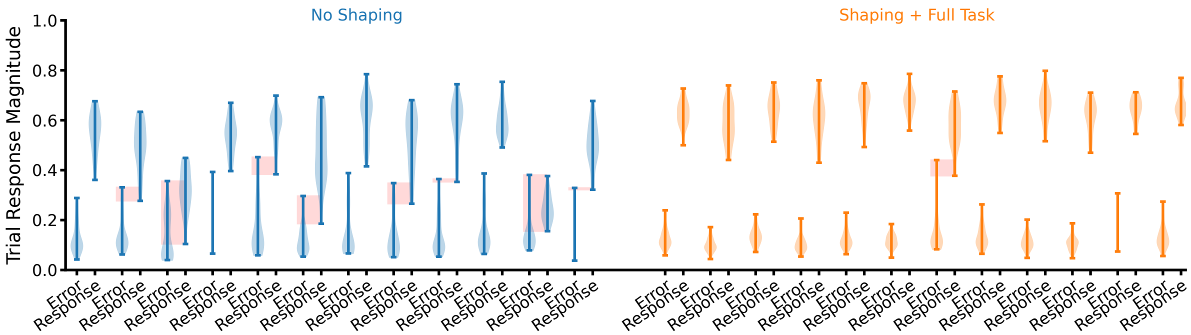

Figure S1. S/FT RNNs more robustly solve the tDNMS task compared to NS RNNs (**a**) Peak values inside the "Error" window compared to the "Response" window during tDNMS task for $n = 12$ NS and $n = 12$ S/FT networks. Following shaping, individual RNNs solve the task robustly for a range of thresholds on the value of the response node, with many RNNs showing an overlap in response magnitudes between the response window and other times throughout the trial (red boxes) or very narrow window of threshold values.

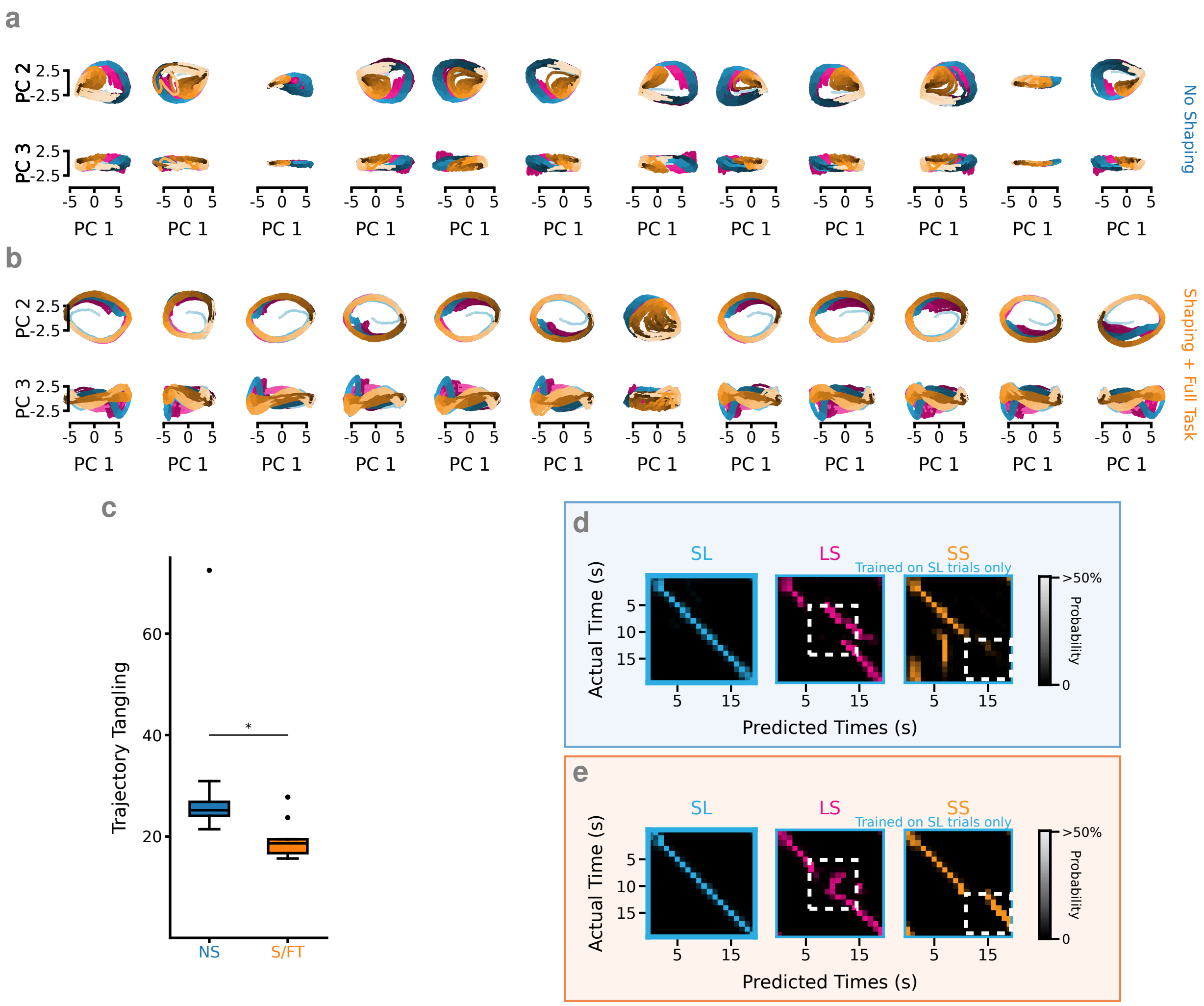

Figure S2. Shaping RNNs constrains network activity to follow smoother state space trajectories. (**a**) PCA state space plots from 12 example NS RNNs independently initialized and trained. *Top*. PC1 and PC2. *Bottom*. PC1 and PC3. (**b**) Same as a but for 12 S/FT RNNs. (**c**) S/FT RNNs show significantly less tangling than NS networks. $n = 12$ NS RNNs, $n = 12$ S/FT RNNs, $p = 0.02$, two sample independent t-test. (**d**) Within- and across-context time decoding for an S/FT RNN from a decoder trained on SL trials. *Left*. LDA based cross-validated decoders trained on SL trials and tested on held out data. *Middle*. The same SL trained decoders tested on LS trials. *Right*. SL trained decoder tested on SS trials. See Figure 2f for LS decoder example. Decoding performed independently and then results averaged across $n = 10$ SL/FT RNNs. (**e**) Same as d but for NS RNN. 2d for LS decoder example.

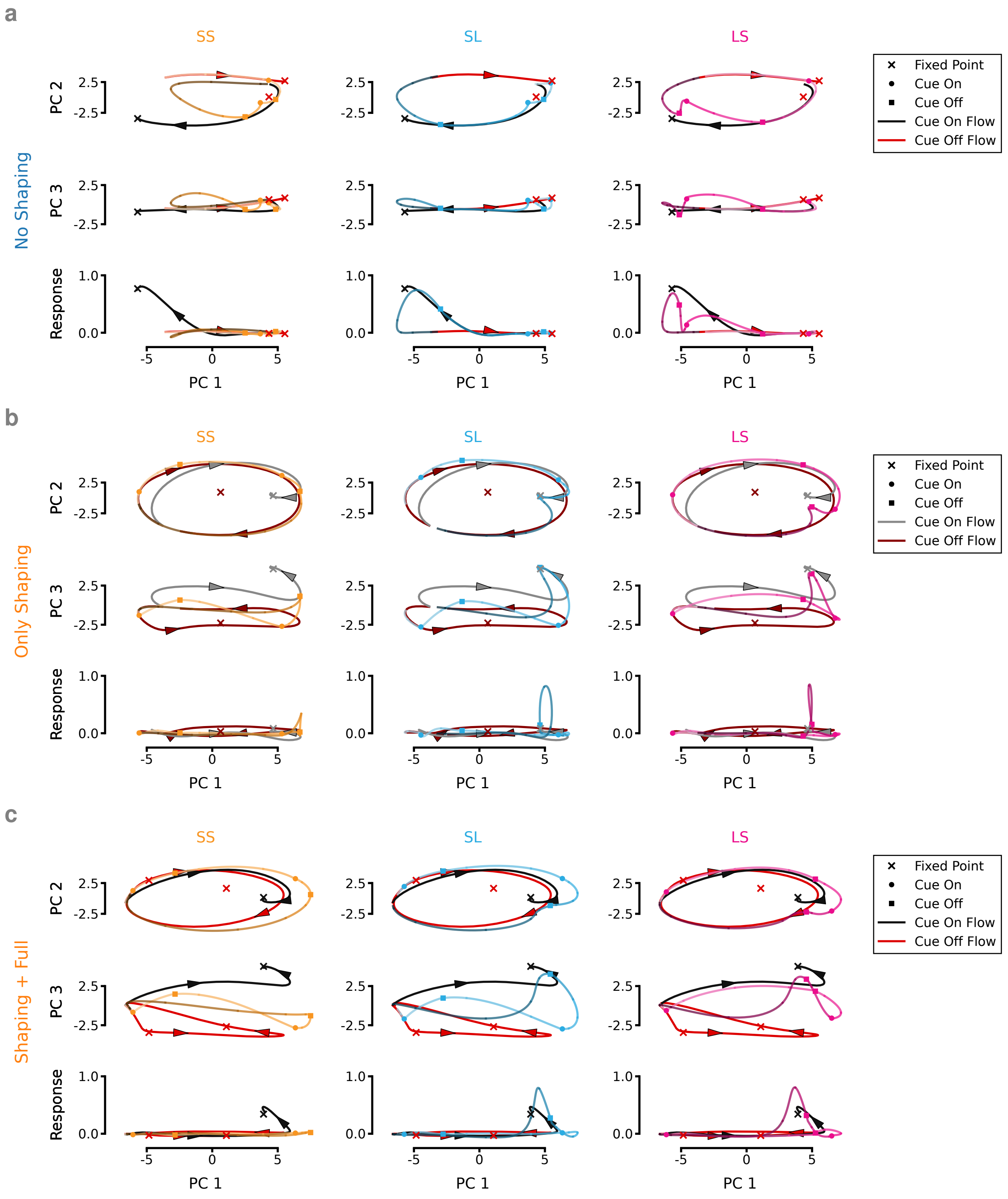

Figure S3. Dynamical motifs reveal how training approach affects RNNs' strategy. (**a**) For each trial type, fixed/slow points for both Cue On and Cue Off modes are shown with state space trajectories superimposed for an NS RNN. *Top*. PC1 vs PC2. *Middle*. PC1 vs PC3. *Bottom*. Response vs PC1. (**b**) Same as a but for an RNN trained on Only Shaping. (**c**) Same as a but for an S/FT RNN.

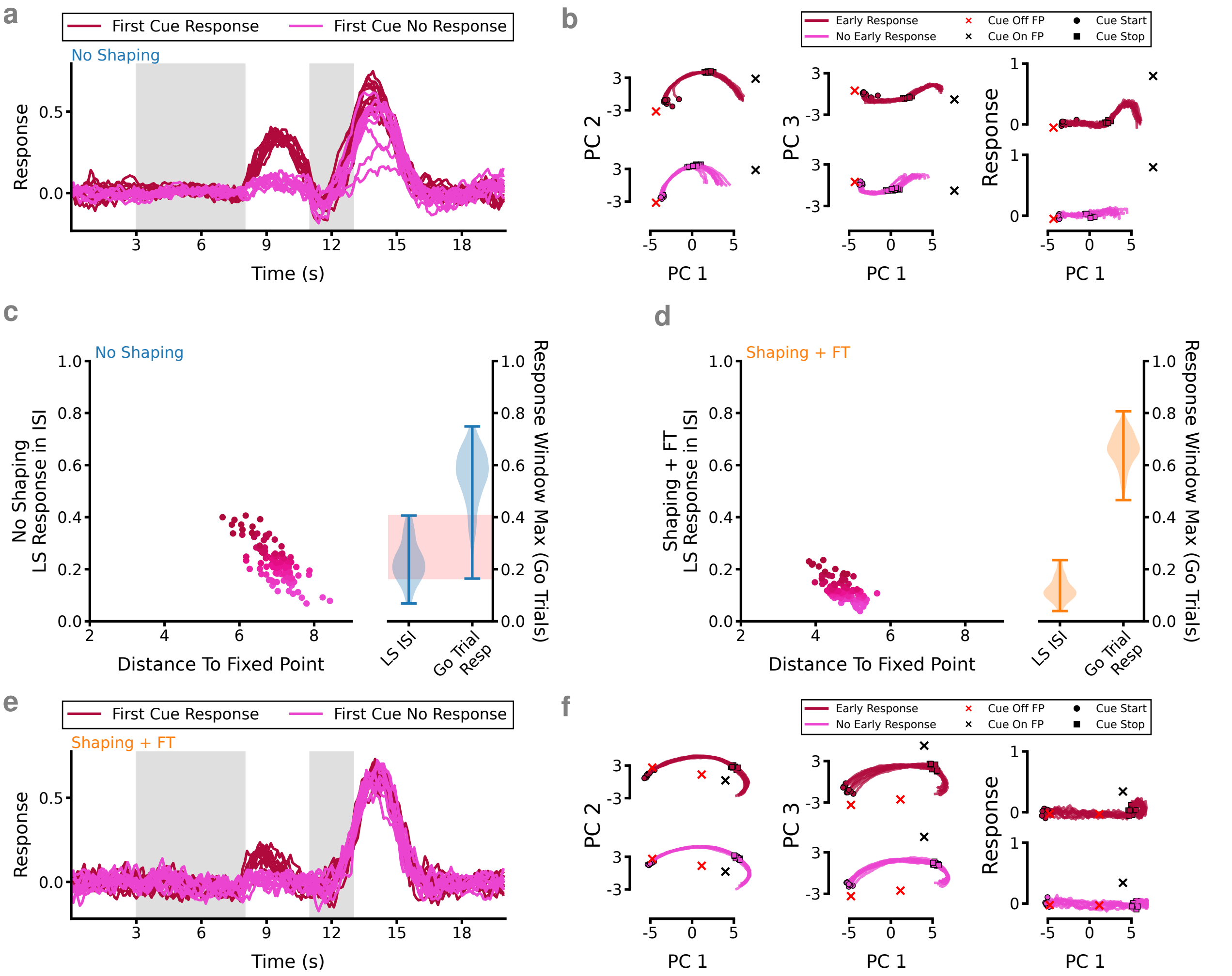

Figure S4. Additional analysis on LS error trials. (a) Comparison of response value between the 10% highest response to the first cue on LS trials (early response error) and the lowest for 1 example NS RNN. (b) *Top*. PCA state space plots spanning the onset of the first cue until the end of the ISI for 10% largest early response trials. *Bottom*. 10% lowest early response. Note trials where the RNN state is closer to the Cue On fixed point when the cue turns off (square) are more likely to exhibit a response. (c) *Left*. Peak response during the ISI compared to euclidean distance to fixed point at the beginning of the ISI shows a strong relationship between how close the RNN is to the fixed point and the likelihood of an early response for $n = 400$ trials in example NS RNN. *Right*. ISI response magnitude peak compared to response window peak magnitude. Overlaps (red) indicates inability to reliably separate “Go” from “No-Go” trials. (d) Same as c but for S/FT RNN. Note there is no overlap in the ISI and response window output. (e) Same as a but for an example S/FT RNN. (f) Same as b but for an example S/FT RNN.

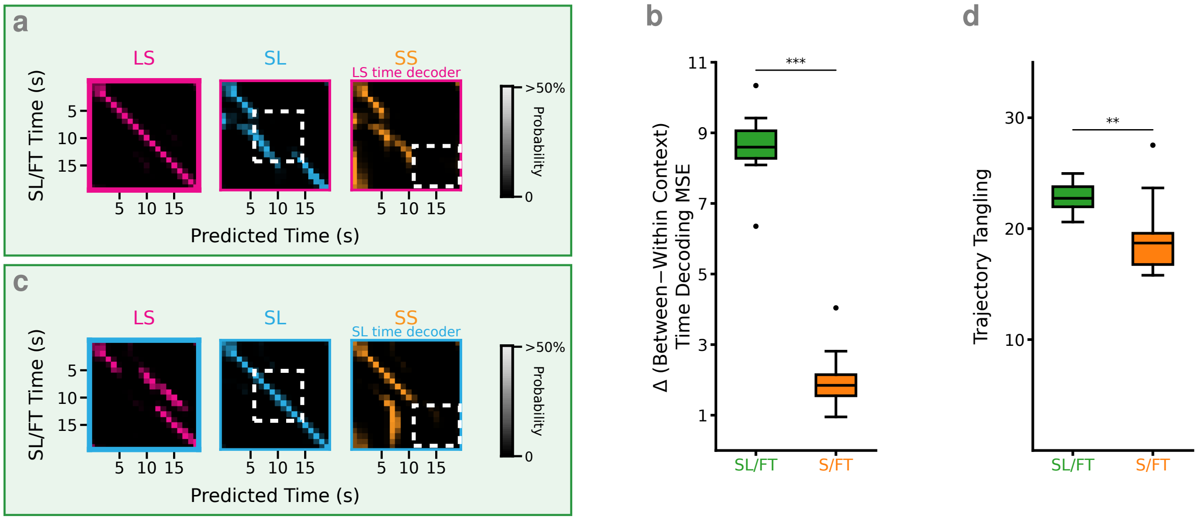

Figure S5. SL/FT networks fail to learn critical abstractions about trial structure. (**a**) Example within- and across-context time decoding for an S/FT RNN. *Left*. LDA based cross-validated decoders trained on LS trials and tested on held out data. *Middle*. The same LS trained decoders tested on SL trials. *Right*. LS trained decoder tested on SS trials. (**b**) Same as a but for decoder trained on SL trials. (**c**) Error comparison for decoding trial time between SL/FT and S/FT RNNs. SL/FT RNNs show decreased performance at cross-context time decoding relative to within, $p = 2.40 \times 10^{-13}$, $n = 10$ SL/FT RNNs, 12 S/FT RNNs. (**d**) SL/FT RNNs show significantly more tangling than S/FT networks. $n = 10$ SL/FT RNNs, $n = 12$ S/FT RNNs, $p = 4.62 \times 10^{-3}$, two sample independent t-test.

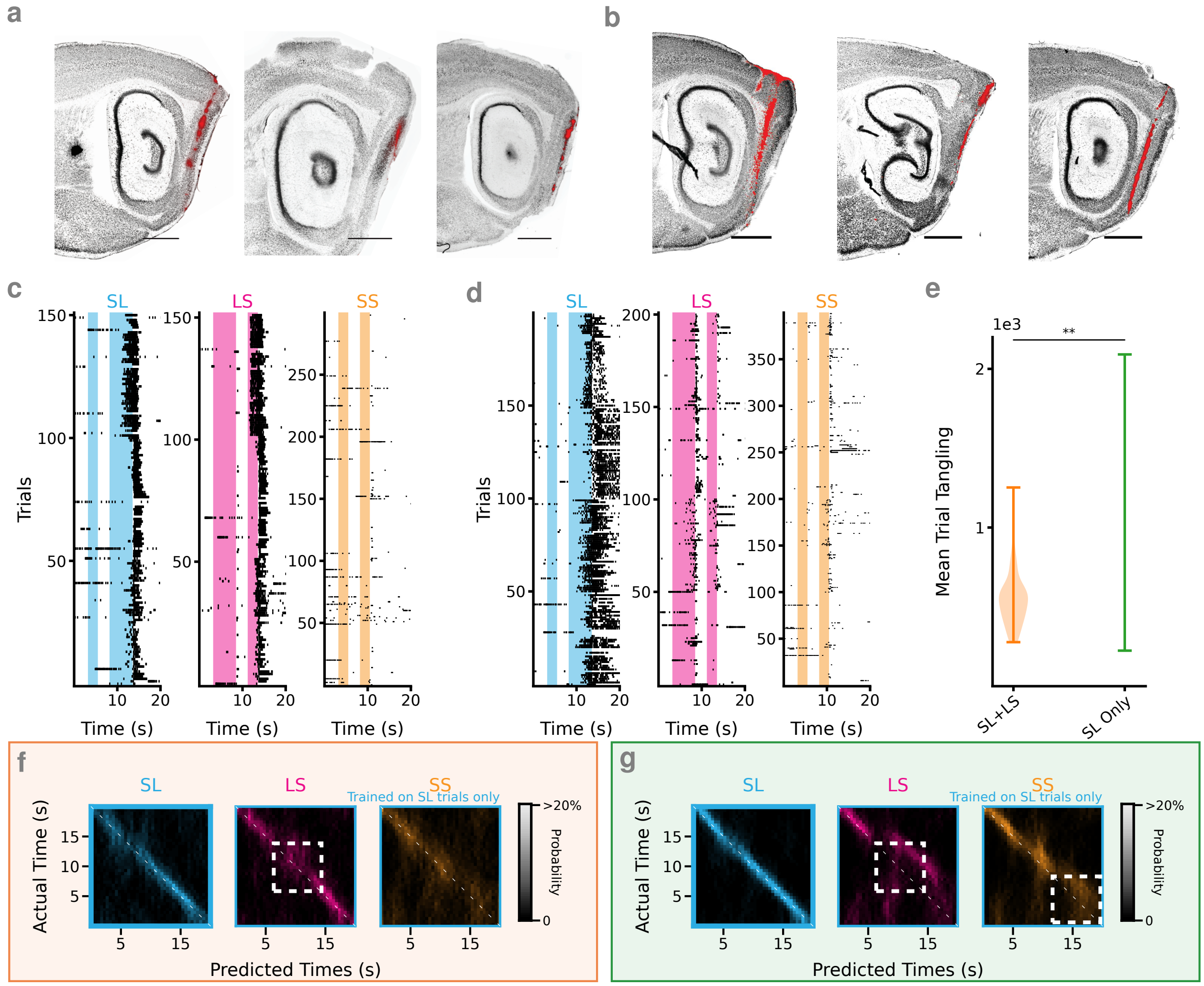

Figure S6. Animals learn shared time-coding axes across trial types—mirroring the dynamics predicted by RNNs. (**a**) Representative section from animal brains trained on S/FT. Nissl stain in black, red dye (DiI/DiD) indicates Neuropixels probe location. Scale bar is 1 mm. (**b**) Same as a but for SL/FT mice. (**c**) Licking activity of all mice trained with SL+LS shaping and then trained on the full task (S/FT mice) on the recording days used for the time decoding analysis. (**d**) Same as c except for the mice trained on SL Only shaping (SL/FT mice). SL/FT mice tend to lick early on LS trials (or not lick at all) resulting in no water reward delivery. (**e**) S/FT shows less tangling than SL/FT (S/FT correct trials only) S/FT $n = 125$ trials from 4 mice, SL/FT $n = 200$ trials from 4 mice, $p = 1.56 \times 10^{-3}$. Tangling caculated on all recorded units across 25 trials per animal. (**f**) Confusion matrix for time decoding on mice trained with LS+SL shaping (10-fold cross validated). Decoder trained on SL trials then tested on LS, SL, and SS trials. Average of $n = 5$ sessions from 4 mice. See Figure 6g for LS decoder. (**g**) Same as g for SL Only trained animals. Average of $n = 6$ sessions from 3 mice. See Figure 6h for LS decoder.

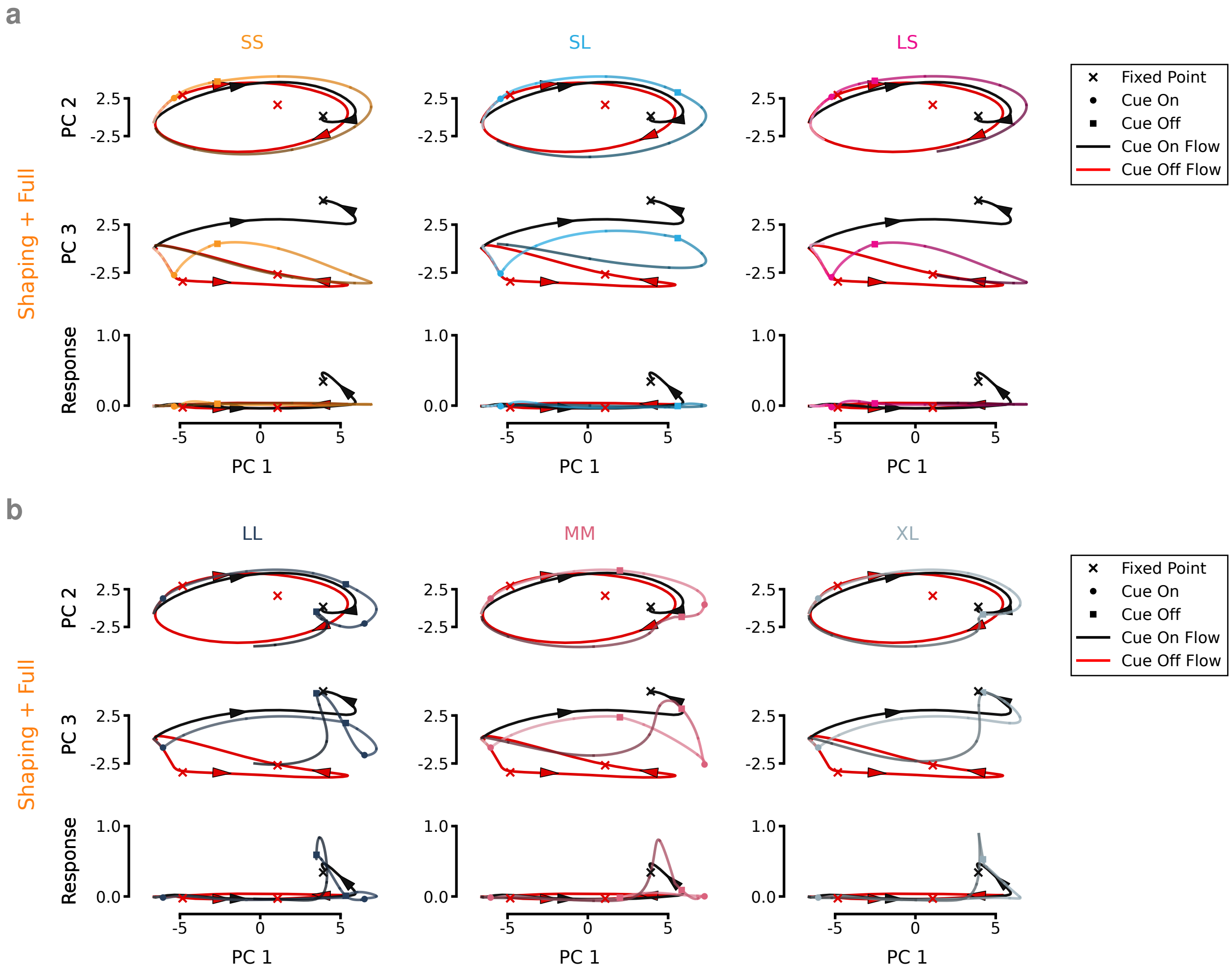

Figure S7. Dynamical motifs make predictions about how RNNs learned strategy extend to novel temporal contexts. (a) Both cues contribute to the RNNs decisions to respond. For each trial type, fixed/slow points for both Cue On and Cue Off modes are shown with state space trajectories superimposed for an example S/FT RNN when the first cue is omitted. Note: SS and LS result is effectively the same in this case. *Top*. PC1 vs PC2. *Middle*. PC1 vs PC3. *Bottom*. Response vs PC1. (b) The strategy adopted by S/FT RNNs predict a response in reaction to several untrained cue configurations. *Left*. Long-Long (LL) trial type comprised of two 5s cues. *Middle*. Medium-Medium (MM) trial type comprised of two 3.5s cues. *Right*. XL trial type comprised of a single 10s cue.
